# 654. Performance of the T2Resistance Panel in Detecting Antibiotic Resistant Bacteria Directly in Whole Blood, and Implications for Improving Appropriate Therapy of Bloodstream Infections

**DOI:** 10.1093/ofid/ofab466.851

**Published:** 2021-12-04

**Authors:** Abigail Skeel, Cornelius J Clancy, Aaron Lucas, Kailey L Hughes, Ryan K Shields, Minh-Hong Nguyen

**Affiliations:** 1 University of Pittsburgh, Pittsburgh, PA; 2 University of Pittsburgh Medical Center, Pittsburgh, PA

## Abstract

**Background:**

Appropriate antibiotic (Ab) therapy of bloodstream infections (BSI) is often delayed by time to blood culture (BC) positivity, species (sp) identification and Ab sensitivity (sensi). The T2Resistance (T2R) Panel is a direct-from-blood (culture-independent) diagnostic that detects 13 genetic markers associated with methicillin-resistant *S. aureus* (MRSA), vancomycin-resistant Enterococcus (VRE), ESBL- and carbapenemase-producing Enterobacteriaceae (E). We assessed T2R performance in detecting these resistant bacteria in whole blood (WB) and analyzed possible impact on time to appropriate Ab.

**Methods:**

We performed T2R using WB samples obtained from patients (pts) on the same day as BCs from July 2019-2020. Receipt of appropriate Ab was assessed at time of empiric, Gram stain-directed, MALDI-directed (sp identification) and sensi-directed therapy. T2R results were not available to care teams. Teams were notified of positive BCs. Stewardship optimized Abs based on sensi.

**Results:**

BC from 103 pts grew 114 bacterial sp: E (n=54; 16 ESBL-, 1 KPC-producer), *S. aureus* (n=29, 22 MRSA), Enterococcus (n=21, 16 VRE), *P. aeruginosa* and others (n=10). 12 ESBL-E produced CTX-M 14/15. T2R sensitivity and specificity was 78% and 99%, respectively, compared to sequencing of resistance markers. Sensitivity was excellent for vanA/B, KPC (100% each), and CTX-M14/15 (92%); sensitivity was 58% for mecA/C. T2R detected resistance determinants in 3-7h. Median time to appropriate Ab was 16.3h, which was significantly longer for VRE (25.6h) and ESBL- or KPC-E (50.9h) BSIs than for T2R marker-negative bacteria (6.7h; p=0.04). Pts with VRE or ESBL-/KPC-E BSI were less likely to received appropriate empiric Ab (18% and 30%, respectively) than pts with T2R marker-negative BSI (63%; p=0.02; Fig.1). Median times to achieve ≥80% appropriate Ab therapy of marker-negative, VRE and CTX-M/KPC-E BSIs were 15.5h (after Gram stain), 43.9h (after MALDI) and 63.5h (after sensi), respectively.

Antibiotic Therapy

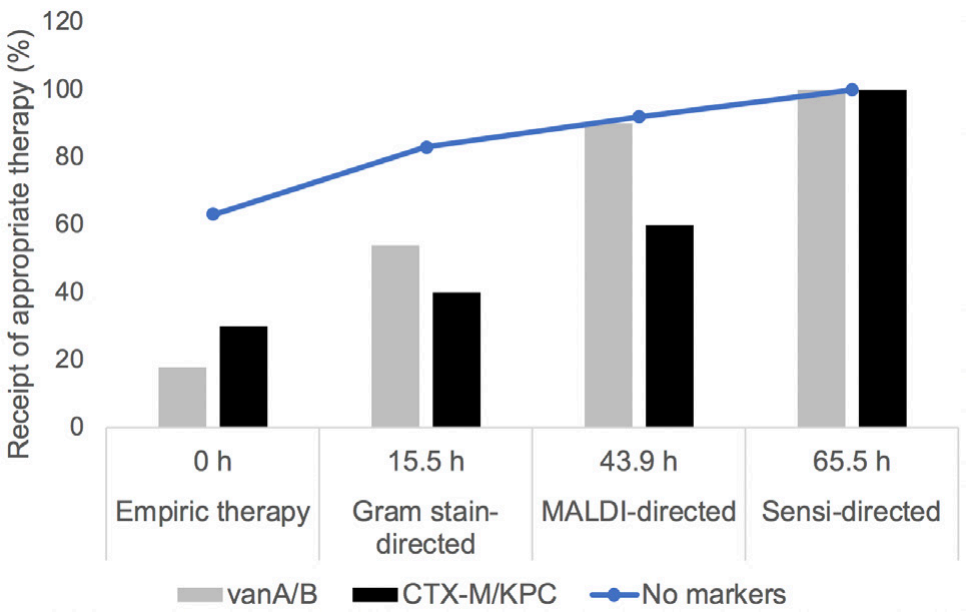

**Conclusion:**

There was a significant delay in appropriate Ab therapy of BSIs, especially in pts infected with VRE and ESBL/KPC-E. T2R rapidly and accurately detected BSI caused by VRE and ESBL/KPC-E, and has the potential to significantly shorten time to appropriate Ab.

**Disclosures:**

**Cornelius J. Clancy, MD**, **Merck** (Grant/Research Support) **Ryan K. Shields, PharmD, MS**, **Shionogi** (Consultant, Research Grant or Support) **Minh-Hong Nguyen, MD**, **Merck** (Grant/Research Support)

